# An Integrative Approach to Promoting Wellness in Older Adults: An Online Undergraduate Course

**DOI:** 10.1093/geroni/igab046.1548

**Published:** 2021-12-17

**Authors:** Robin Majeski

**Affiliations:** University of Maryland, Baltimore County, Catonsville, Maryland, United States

## Abstract

As gerontology and geriatric programs look to grow their connections with health and allied health areas, examining emerging health care areas is important. Thus, this session will present key concepts and pedagogical strategies from an innovative online undergraduate course, An Integrative Approach to Promoting Wellness in Older Adults, that is taught in the Erickson School at the University of Maryland at Baltimore County. The course brings together a conventional western allopathic approach and a holistic, patient-centered, integrative approach which includes complementary therapies, to promote wellness in older adults. Specifically, the session will present a comparison of the philosophical premises of conventional western and integrative approaches to health promotion. It will present a holistic model of health, an overview of the effectiveness and safety of different complementary therapies, and examples of how an integrative approach with conventional western and complementary therapies can be used in to promote health in older adults with particular chronic conditions. Also, a discussion of the incorporation of diversity content in the course, an integrative approach to health promotion for underserved populations, and specific pedagogical strategies used to facilitate learning in synchronous and asynchronous online learning environments are included. Session presentation techniques include a PowerPoint presentation, demonstration of examples of conventional western and integrative approaches to health promotion in older adults through case studies, and audience participation.

